# Design and implementation of magnetically–tunable quad–band filter utilizing split–ring resonators at microwave frequencies

**DOI:** 10.1038/s41598-020-57773-6

**Published:** 2020-01-23

**Authors:** Cihan Asci, Aydin Sadeqi, Wei Wang, Hojatollah Rezaei Nejad, Sameer Sonkusale

**Affiliations:** 1 Nano Lab, Advanced Technology Laboratory, 200 Boston Ave, Medford, MA 02155 USA; 2 Tufts University, Department of Electrical and Computer Engineering, 161 College Ave, Medford, MA 02155 USA

**Keywords:** Engineering, Electrical and electronic engineering

## Abstract

In this article, we present a magnetically–tunable quad–band filter with high tunability in the frequency range of 2.1–3.9 GHz. A multi–band filter with four stop–bands comprises of a microstrip line coupled to four frequency–selective split–ring resonators (SRRs). We achieve tuning of individual frequency bands using magnetic reed switches connected in between the capacitive gaps of each split–ring resonator. Application of magnetic field tunes this capacitance affecting its resonance frequency. The measured reflection spectrum of the proposed device matches well with the simulation results. The results show more than 25% tunability for each of the four bands with bandwidth values in the range of 30–70 MHz with over 100% overall tunability in the 2.1–3.9 GHz frequency spectrum.

## Introduction

Metamaterials are artificially engineered structures that can be designed for achieving tailored electromagnetic properties that do not occur naturally^1^. In this respect, there has been a growing interest on the design of different metamaterials over the past couple decades. Key applications of metamaterials include the design of an effective medium with a negative magnetic permeability, perfect lens, perfect absorbers or imagers^2–5^. The ability to manipulate and control permeability and permittivity in metamaterials has been used to realize innovative microwave and millimeter–wave circuits and sensing platforms^6–16^. Hence, metamaterials can also be utilized in microwaves and antenna systems^17–22^. Yet some other applications where tunable metamaterials have been realized and applied are in the area of optical or plasmonic metamaterials with applications in biosensing^23^ and optics^24,25^. In that sense, plasmonic metamaterials can be implemented in order to get negative refraction and negative refractive index^26,27^. Metamaterials are built using individual resonators of which split–ring resonators (SRRs) are one of the most commonly used. Adjusting the dimensions such as the length and the gap of the SRR enables one to realize metamaterials with resonances from microwave to optical frequencies. Moreover, one can actively tune the resonance frequency by incorporating a tunable circuit element such as a transistor, diode or a varactor^5,28,29^.

Emerging new standards and demand for wireless communications have necessitated transceivers that can work across multiple frequency bands. A key element in such transceivers is compact multi–band filters with an ability to support multiple standards^30^. Circuit elements such as diodes, varactors, MEMS switches have been demonstrated for frequency tuning of filters; however, such filters support only a few bands with limited tuning range or exhibit high insertion loss^31,32^. CMOS–based active switches, diodes and varactors suffer from intermodulation and harmonics due to the undesirable nonlinear properties of such active devices at high power and high frequencies. Moreover, active switches have off–state leakage, and are sensitive to the process–voltage–temperature (PVT) variations resulting in unreliable implementations. In fact, PVT variations are expected as in any other filters, and are not unique to this realization. Hence, active switches realized by MOS transistors are affected by these variations. For instance, variations in MOS transistor dimensions (W/L ratio) are subject to the photolithographic processes. Temperature fluctuations due to power dissipation are also effective on the transistor operation in which the power consumption is mainly due to the switching and leakage. Thus, there is a need for achieving a better switch immune to the PVT variations with low–leakage current under off–state.

In this paper, low–loss magnetic reed switches have been embedded into the filter structure to achieve low–leakage magnetically–tunable capacitors/switches to tune the resonances and thus the frequency bands. Reed switches comprises of a pair of flexible metal reeds with the end terminals that are normally separated by a small gap when the switch is open. Two reeds are sealed within a tiny tubular, and the magnetic field present nearby leads to the two reeds connected to close the switch. Increasing the magnetic field during the off states causes the two metal reeds to come closer increasing the effective capacitance and thus provide a magnetically–tunable capacitor. Conventional realization of reeds switches since the 1950s^33^ have been replaced with MEMS–based realization where smaller footprint and integration is desired^34^. However in this work, for proof of concept, a bulkier implementation was adequate since our feature sizes for the filters were sufficiently large. Future realizations can incorporate MEMS based reed switches for higher frequency and more compact realizations.

**Figure 1:**
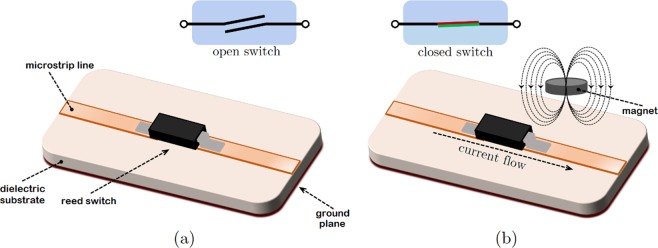
Working principle of the SPST normally–open (NO) reed switch. (**a**) Open condition (off switch). (**b**) Closed condition (on switch) in the presence of a magnetic field.

A reed switch is a passive circuit component that does not suffer from the nonlinearity of the active switch counterparts mentioned before. Due to two separated reeds, it possesses an extremely low leakage current during off–state. In the light of these properties, magnetic reed switches are proposed in this work to turn on and off the particular resonance (notch) frequency of the stop–bands. One can also tune the frequency in analog fashion by adjusting strength of the magnetic field during the off–state that controls the effective capacitance of the reed switch. The basic reed switch structure is the single–pole single–throw (SPST) normally–open (NO) switch that is also known as Form A switch. Other form types are available such as Form B and Form C where the switches are SPST normally–closed (NC) and single–pole double–throw (SPDT) also known as changeover, respectively^35^. Figure 1 summarizes the architecture and working principle of the SPST NO reed switch. As it can be seen from the figure, when there is no magnetic field around the reed switch, there is no electrical connection. Once a sufficient magnetic field is applied to the switch, one is able to close the switch resulting in an electric current to flow between the terminals of the reed switch and the system that the switch is connected to.

**Figure 2:**
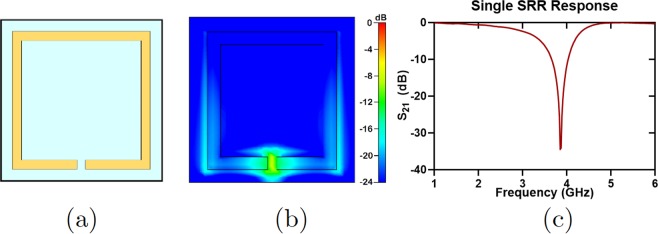
The rectangular single–split resonator used in the quad–band notch filter. (**a**) The resonator used in the filter with the largest dimensions. Other resonators will be analyzed in the next section. (**b**) Electric field confinement of the SRR when tested with waveguide ports in CST/MWS. (**c**) The resonance of the single–split SRR around 3.85 GHz.

A single—split rectangular resonator is shown in Fig. 2(a). An incident electromagnetic wave on this SRR with an E–field along the capacitive gap results in confinement of an electric field in the gap region at its resonance frequency as shown in Fig. 2(b). However, SRRs are not restricted to only the rectangular geometry^36–39^. In this work, the rectangular single—split resonator is chosen because of its relative simplicity of fabrication. The resonator shown in Fig. 2 has been designed by changing its lateral dimension while the other dimensions such as conductor width and gap width are kept constant. The electric field confinement can be observed in the gap region at the resonant frequency of 3.85 GHz. The critical dimensions of all resonators utilized in the quad–band filter will be summarized in Table 1 in the next section. One can tune the resonance frequency by placing a circuit element in the capacitive gap of the SRR. In this work, we utilized magnetic reed switches as active circuit tuning element.

**Table 1:** Dimensions of quad–band filter structure. All units are in mm.

Variable	Value
*W*	0.75
*W*_50_	3.51
*W*_*s*_	35
*L*_*s*_	15
*g*	0.5
*h*	0.65
*L*_1_	3
*L*_2_	4.5
*L*_3_	6
*L*_4_	7.5
*L*_5_	7

## Filter Structure and Design

**Figure 3:**
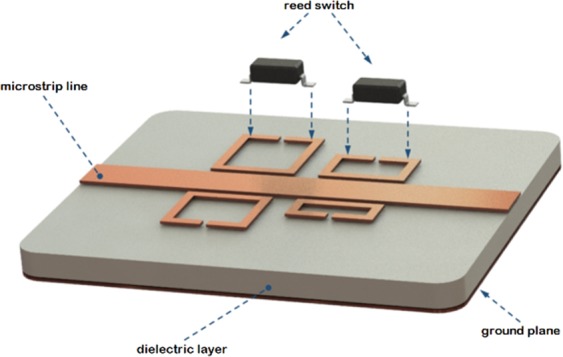
3D view of the magnetically tunable quad–band microwave filter. Each resonator has a magnetic reed switch connected across its capacitive gap. Only two switches are shown for clarity.

A quad–band filter designed to operate at microwave frequencies between 2.1–3.9 GHz is implemented and consists of a microstrip line coupled to four different SRRs each with unique resonant (notch) frequency. Magnetic reed switches are present across each of the capacitive gaps of the SRR. The implementation comprises of Rogers RO4003C substrate from Rogers Corporation (Chandler, AZ, USA) with a height of 1.524 mm (60 mil) and a copper trace thickness of 0.017 mm (0.7 mil, 0.5–oz Cu) as shown in Fig. 3. In the filter topology, split–ring resonators are coupled with the 50Ω–microstrip line such that the device can resonate at different frequencies. The substrate material has a dielectric constant of 3.38, and hence, with these substrate board material parameters, width of the 50Ω–microstrip line is calculated as 3.51 mm. With the utilization of reed switches, these resonant frequencies can be tuned to higher or lower frequencies in the vicinity of a magnetic field.

**Figure 4:**
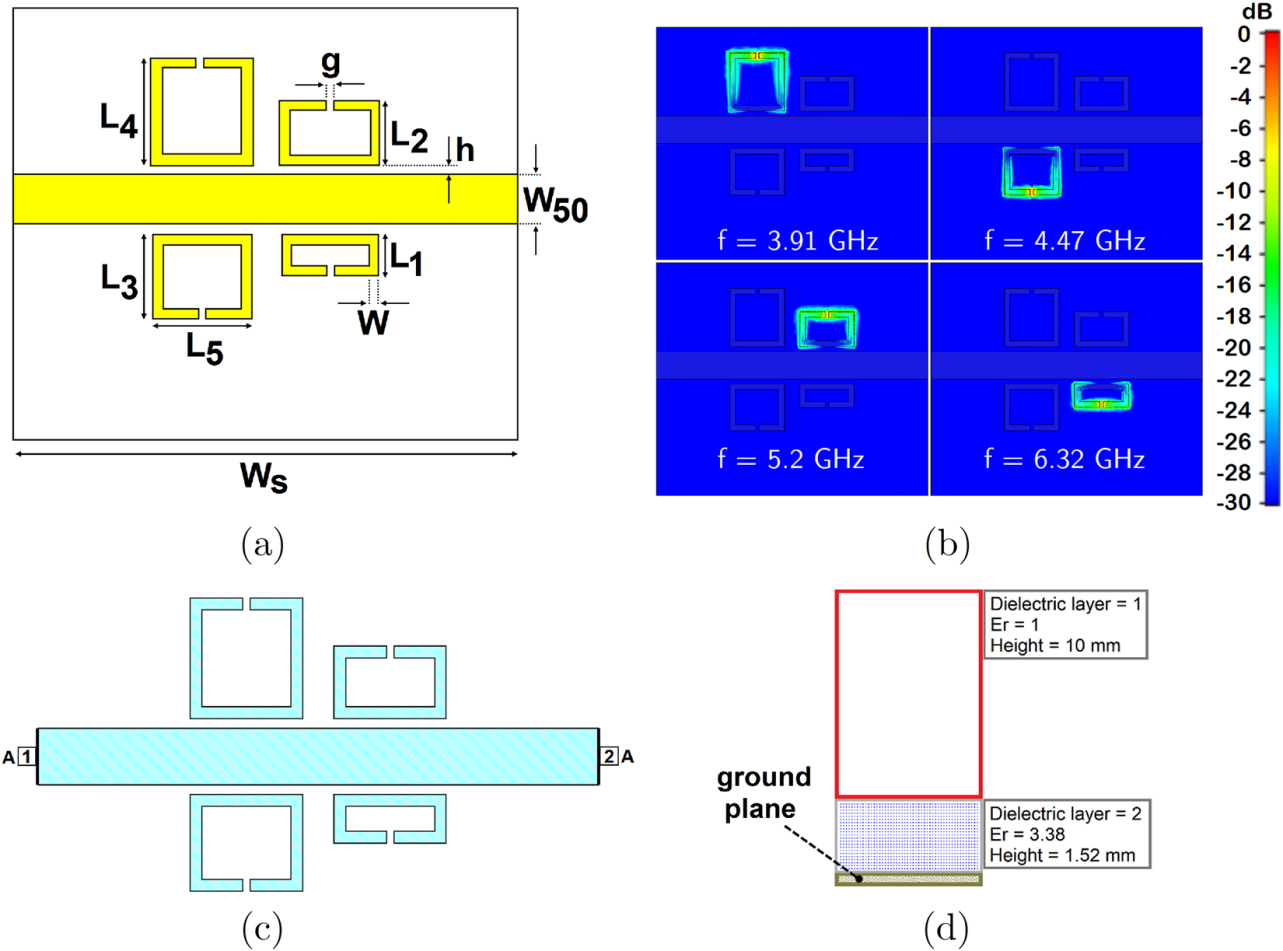
Quad–band filter structure and simulation results: (**a**) Top view of the filter with the critical dimensions. (**b**) Electric field confinement of the device at resonance frequencies. E–field results are obtained in CST/MWS. The resonant frequency values of SRRs are 3.9, 4.4, 5.1, and 6.2 GHz. (**c**) The layout of the quad–band filter simulated in AWR/MWO with two ports and a grid size of 0.05 mm for the EM analysis. Light blue areas indicate the conductors laid on the Rogers RO4003C board. Auto ports are denoted as “A” at the input and output of the filter. (**d**) Stackup used in AWR/AXIEM where the bottom layer is defined (olive color) as ground plane and the air region (enclosed between red lines).

**Figure 5:**
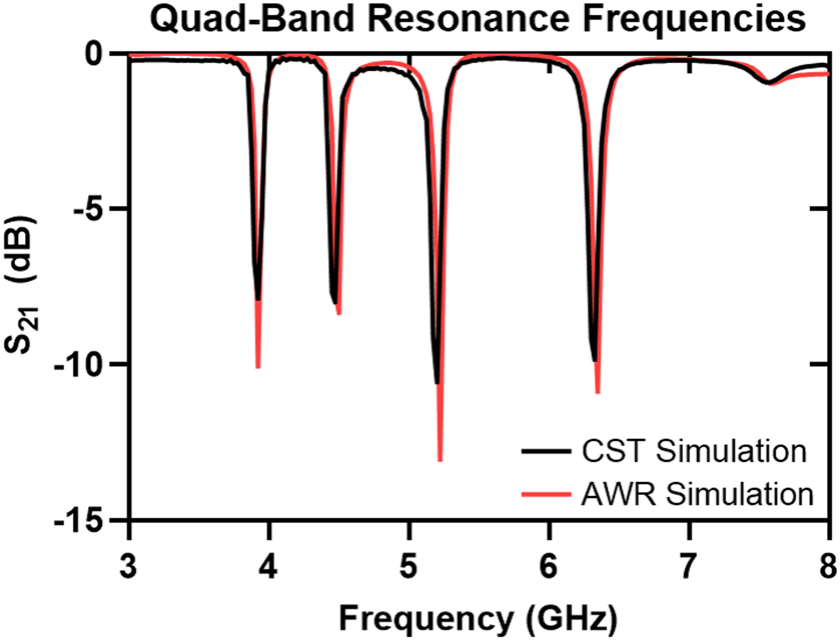
Resonant frequencies of the quad–band filter structure given in Fig. 4(a). Solid green line and black dotted line show the CST/MWS and AWR/MWO simulation results, respectively.

Split–ring resonators and filter structures are modeled and simulated using commercially available electromagnetic (EM) simulation software: CST Microwave Studio (CST GmbH, Darmstadt, Germany), and AWR Microwave Office (AWR Corporation, El Segundo, CA, USA). First, the quad–band filter structure has been designed without using reed switches as shown in Fig. 4(a). In the simulation, the filter has been fed by two wave ports at the end of each microstrip line. Open boundaries are defined in CST design environment because only port characteristics (*S*_11_ and *S*_21_) and electric field confinement of each SRR have been analyzed. For the broadband sweep of the filter structure, the frequency–domain solver that assumes a time–harmonic dependence of the fields in Maxwell’s equations has been used. Adaptive tetrahedral mesh refinement is utilized for creating a better mesh structure for the problem. The normalized electric field confinement of each resonator at their corresponding frequencies is shown in Fig. 4(b). As the lateral dimension of a resonator in this topology increases, the resonant frequency decreases. Different resonant frequencies for different resonators have been achieved by changing only the lateral dimension of the resonator while all other physical parameters are kept constant. The designed single–split resonators show resonances at 3.91, 4.47, 5.2 and 6.32 GHz. Without using reed switches, the device given in Fig. 4(a) is a band–stop filter without any tuning property, and therefore, the insertion loss of the filter has been obtained using CST/MWS and AWR/MWO in order to validate the notch filtering operation at the frequencies given above. Figure 5 shows the notch bands for both AWR and CST simulation cases in which the discrepancy between them is very small. It is plausible to expect such small differences in these simulations because the EM solvers in both softwares are different; CST is based on finite integration technique (FIT) method while AXIEM, a planar 3D EM simulator in AWR/MWO, is based on method of moments (MoM). However, for both simulations, mesh optimization and adaptive mesh refinement in all frequencies have been utilized, and thus, for different solvers, similar responses have been observed. The layout of the quad–band filter drawn in AWR is given in Fig. 4(c). In 3D full–wave simulation of the filter structure in CST/MWS, wave port excitations have been used; however, in AWR/MWO, the signal excitation ports are defined as auto ports that are self–configurable ports based on the stackup shown and geometry of the given problem^40^. The stackup for the analysis of the quad–band notch filter is shown in Fig. 4(d).

For multi–band operation, more resonators can be added to the structure so that the added SRRs can couple to the propagating wave in the microstrip line creating different resonant frequencies and corresponding stop bands in the transmission spectrum. Also, in order to attain lower resonant frequencies due to the SRRs, single–split structures with larger lateral dimensions can easily be added to the filter topology; one may need to adjust the dimensions of the RF board to accommodate additional resonant frequencies (or bands).

## Experimental Results

**Figure 6:**
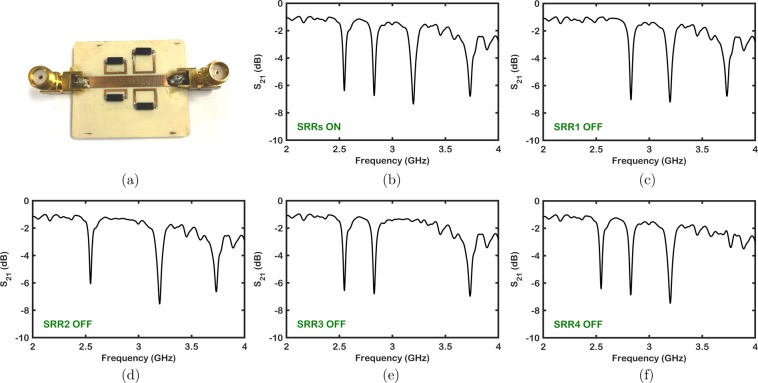
(**a**) Fabricated quad–band magnetically–tunable microwave filter. Reed switches are soldered on the gap of each resonator. (**b**) All resonators are in ON state. Through (**c**) to (**f**), SRR1, SRR2, SRR3 and SRR4 are in OFF state, respectively.

Magnetically tunable quad–band microwave filters comprised of a microstrip line, split–ring resonators and reed switches have been implemented on commercially available double–sided Rogers RO4003C high–frequency boards patterned using LPKF Protomat S103 (LPKF Laser & Electronics AG, Garbsen, Germany) with standard printed circuit board (PCB) fabrication methods. After milling the board, magnetic MEMS reed switches (MK24–A–3, a SPST normally open (NO) switch from Standex Electronics, Cincinnati, OH, USA) are soldered in the gap of each individual single–split resonator as seen in Fig. 6(a). Two important performance parameters of switches are release time and operating time. The operating time can be defined as the transition time between the moment when the voltage is applied to the relay coil to the time instance when the NO switch contacts close. Hence, it is the time interval between the initial application of power to the coil and the closing of the normally–open contacts. On the other hand, the release time is defined as the time interval between the removal of the power applied to the coil to the reopening of the closed contacts. In the datasheet of the reed switch, the operating time and release time are provided as 0.25 ms (max), and 0.15 ms (max), respectively^35^. These values indicate that the selected reed switch performs reasonably in terms of switching response. It is measured (with Magnetometer Model MR3 by AlphaLabs Inc., Salt Lake City, Utah, USA) that a magnetic field intensity of 1057 mGauss should be applied to the reed switch on average in order to initiate the actuation for switching.

**Figure 7:**
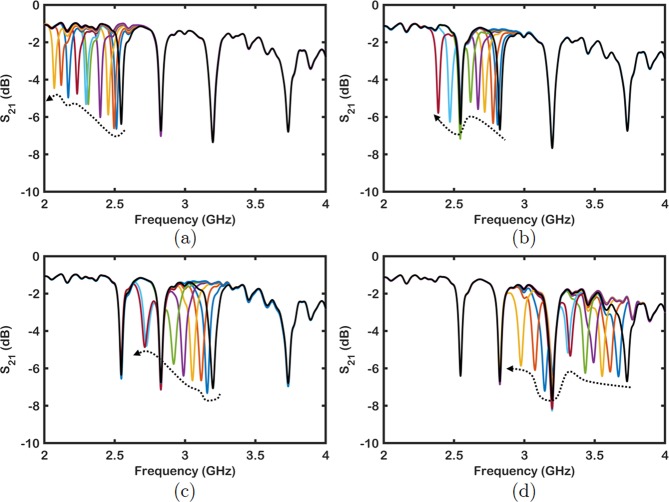
Frequency shift of each resonator by changing the magnetic field intensity near the reed switch. (**a**) Frequency shift in resonator 1. (**b**) Frequency shift in resonator 2. (**c**) Frequency shift in resonator 3. (**d**) Frequency shift in resonator 4.

The insertion loss (*S*_21_) characteristics of the microwave filters have been measured using N5227A PNA Microwave Network Analyzer (Keysight Technologies, Santa Rosa, CA, USA) shown in Fig. 7. When all the reed switches are ON, the notch bands of the quad–band filter are located at 2.55, 2.83, 3.2 and 3.7 GHz. The discrepancy between the simulated filter structure without reed switches and measured filter response is due to the fact that reed switches add parasitic effects. The quad–band filter is tuned by applying magnetic field individually to each reed–switch with its own micro–coil inductor that will adjust the magnetic field by programming the current flowing through its coil. Microcoils with their own current control can be as small as a single loop of wire with a current passing through it. If manual control is needed, one can also use Neodymium magnets that are local to each reed switch. Therefore, each resonator can be turned ON or OFF by selectively changing the magnetic field around the corresponding reed switch. As shown in Fig. 6(b), when all the resonators are in the ON state when there is no change in the magnetic field. It is possible to turn ON and OFF each individual resonator as presented in Fig. 6(c–f) as well as to control multiple resonators simultaneously.

Due to the fact that the reed switches are sensitive to magnetic field intensity, the resonant frequency of each notch band can be tuned to different frequency values instead of having only ON and OFF states. All resonators with reed switches show tunability through change in its capacitance by adjusting magnetic field intensity under OFF–state as seen in Fig. 7. The bands from lower to higher frequencies, each notch band has a bandwidth of 32, 34, 47 and 73 MHz, respectively. The insertion loss of each in the passband is less than 2.5 dB for four notch bands in 2.0–3.7 GHz frequency range. As seen from Fig. 7, the colored lines indicate that each band is tunable over 25% before the corresponding resonator turns OFF implying that the composite filter has an overall tunability greater than 100%.

**Figure 8:**
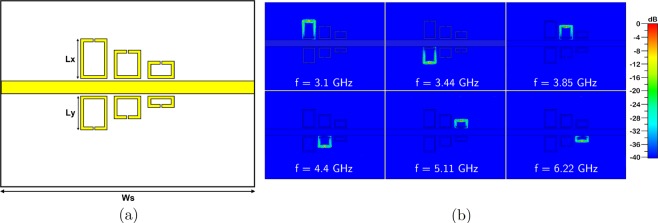
Quad–band filter structure is extended to six tunable frequency bands. (**a**) Top view of the multi–band filter with critical dimensions *L*_*x*_, *L*_*y*_ and *W*_*s*_. (**b**) Simulated electric field confinement of each resonator.

**Figure 9:**
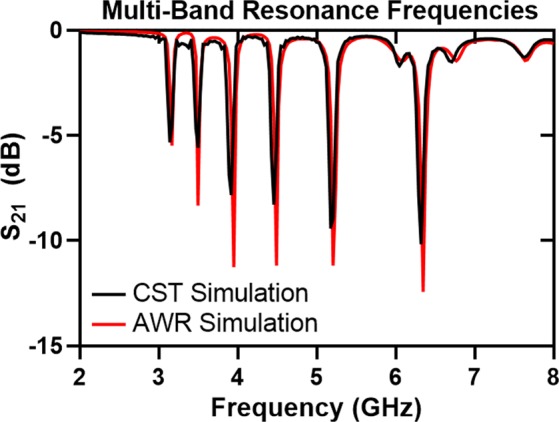
Insertion loss simulations of the filter structure with increased number of notch bands have been performed using CST/MWS and AWR/MWO.

The number of tunable bands that can be achieved with reed switches and microstrip structure is not limited to four as presented in this work. More frequency bands can be obtained by adding more single–split resonators to the structure such that the resonant frequencies of each individual structure correspond to their own bands. As shown in Fig. 8(a), two more resonators are added to get more notch bands at lower frequencies. CST/MWS simulation shows that the added in simulation single–split structures resonate at 3.1 and 3.44 GHz. Electric field confinement of the designed split–ring resonators are shown in Fig. 8(b). Vertical dimensions of the larger resonators are denoted in the figure as *L*_*x*_ and *L*_*y*_ with values of 10.5 mm and 9 mm, respectively. The board width Ws is also changed to 68 mm. All other dimensions stay the same as with the quad–band tunable filter. The insertion loss of the filter shown in Fig. 8(a) has six notch bands in the frequency spectrum as shown in Fig. 9. We performed *S*_21_ simulations in CST/MWS and AWR/MWO in order to complement simulation and experimental results for the quad–band filter.

There may be difficulty to realize much higher tunable notch frequencies. In that case, the geometrical structure of single–split resonators will be very small on the size scale of the reed switches themselves. In order to overcome this difficulty, reed switches can be placed on the back–side of the circuit board and connected to the gap region using standard vias. Although it might seem simple and effortless to integrate many reed switches on the back side of the circuit board, using standard vias will introduce additional capacitances affecting the resonant frequencies of each band. However, the adverse effect can be minimized with the design of the circuit board using full–wave simulations. Additionally, a sandwich–type structure that has a micro–coil array might be held in the close proximity to the top layer of the filter board so that the inductances of each coil can be tuned electronically. In this respect, analog frequency tuning of notch bands might be achieved. These modifications including arrayed control of the resonators using micro–coil array will be focus of the future work. While stop–bands (notches) have been shown here, one could also implement magnetically tunable multi–pass–band filters.

## Conclusion

In this paper, a magnetically–tunable, quad–band band–stop filter has been designed, implemented and demonstrated. The primary component of this microwave filter is a magnetic reed switch that is a magnetically controllable normally–on (NO) SPST switch and utilized for turning on and off of the each split–ring resonators. An analog tuning during off–state is also demonstrated by controlling the strength of the magnetic field which tunes its capacitance. The application of magnetic fields to individual reed switches connected to different split–ring resonators can be used to cause a frequency shift or complete disappearance of notch bands based on the applied magnetic field intensity. This work delivers a compact tunable band–stop filter comprised of split–ring resonators and reed switches in between 2.1–3.9 GHz with more than 25% tunability for each notch band, while making the composite filter structure with an overall tunability of 100%.
